# Practice nursed-based, individual and video-assisted patient education in oral anticoagulation - Protocol of a cluster-randomized controlled trial

**DOI:** 10.1186/1471-2296-12-17

**Published:** 2011-04-10

**Authors:** Thanh Duc Hua, Stefan Viktor Vormfelde, Manar Abu Abed, Hannelore Schneider-Rudt, Petra Sobotta, Tim Friede, Jean-François Chenot

**Affiliations:** 1 Department of General Practice, University Medical Centre Göttingen, Humboldtallee 38, Göttingen, 37073, Germany; 2 Department of Clinical Pharmacology, University Medical Centre Göttingen, Robert-Koch-Str. 40, Göttingen, 37075, Germany; 3 Department of Medical Statistics, University Medical Centre Göttingen, Humboldtallee 32, Göttingen, 37073, Germany

## Abstract

**Background:**

Managing oral anticoagulant treatment (OAT) is a challenge for patients and primary care providers. It requires a high level of patient knowledge and adherence. Studies have shown that insufficient adherence and a low level of patient knowledge about OAT are primary causes for complications. This trial is the first to evaluate the long-term effects of a complex practice nurse-based patient education program in comparison to a patient brochure only.

**Methods and design:**

This trial will be a cluster-randomized controlled trial in 22 general practices (GPs) recruiting 360 patients with OAT. GPs will be randomized into an intervention group or a control group. A baseline questionnaire will assess pre-existing knowledge about OAT. The patients in the intervention group will be educated by a complex education program which consists of a video, a brochure and individual training by a practice nurse. The video gives information about OAT, nutrition, and instructions about how to manage critical situations. The brochure repeats the content of the video. After 4 to 6 weeks, the intervention will be recapitulated. The control group will receive the brochure only. After 6 months, questionnaires will be used in both groups to assess patient knowledge about OAT as well as patients' subjective feelings of safety. Separately, we will evaluate patient records, looking for documented complications and the time spent in the therapeutic range.

**Discussion:**

This trial will start in January 2011. This trial will evaluate the long-term effectiveness of a video-assisted education program on patients with OAT in comparison to a patient information brochure. Most previous studies have evaluated knowledge directly after an educational intervention. Our trial will look for long-term differences in basic knowledge of OAT. We expect that our complex patient education program effectively increases long-term basic knowledge about OAT. Although the population of our study is too small to observe differences in adverse effects, we expect to discover differences in secondary outcomes, such as the time spent in the therapeutic range.

**Trial registration:**

Deutsches Register Klinischer Studien (German Clinical Trials Register): DRKS00000586

Universal Trial Number (UTN U1111-1118-3464)

## Background

Managing oral anticoagulant treatment (OAT) is a challenge for patients in their daily life. In Germany, more than a half million patients are taking phenprocoumon daily and this number will increase in the next few years due to the demographic development, as in other industrialized countries. The incidence and prevalence of atrial fibrillation (AF), the major indication for oral anticoagulation, increases progressively with age, and approximately 50% of patients with AF are 75 years or older. With the aging of population, the number of adults with AF and OAT will increase markedly over the next several decades [1]. Intra- and inter-individual variability of the dose-effect relationship is affected by several factors such as patient adherence, nutritional patterns, genetic differences and drug interactions [2-4]. Serious complications arise from insufficient or excessive anticoagulation. Main complications of OAT are bleeding complications, which occur in 0.3 to 0.4% of all patients with OAT every year [5]. Unlike in many other countries, in Germany phenprocoumon is the most commonly-used drug for OAT. An accurate estimation of the complication rate in association with phenprocoumon does not exist. An English study assessed an annual bleeding risk of 9.0% for Warfarin which is usually prescribed in USA and the United Kingdom [6].

Lack of patient education, misjudgements of the indication, incorrect and misleading documentation, insufficient communication and cooperation as well as lack of patient's integration into the therapy are common causes for an unsatisfactory management of OAT [7]. Studies have confirmed that insufficient adherence and a low level of patient knowledge about OAT are the primary causes for complications [7-9]. Therefore, it is necessary to increase patient knowledge. Patient self-management (PSM) of international normalized ratio (INR) shows that intensive obligatory training lowers the risk for severe bleeding complications [10-12]. A systematic review concluded that PSM is not feasible for many patients because of patients' inability to complete training [13,14]. Another barrier for implementation of PSM is costs [15]. A standardized education program in primary care for patients who are not suitable for PSM is lacking in many countries [7,9]. The best strategy for educating patients about oral anticoagulation has not been determined yet [16]. In Germany, general practitioners manage patients with OAT. Some general practitioners use self-written patient information brochures to inform patients about OAT. However, the contents and quality of these brochures can differ greatly and sometimes even contradict evidence from published literature.

So far, an Italian study has evaluated the short-term effects of an education program which was well accepted by the patients but did not improve the time spent in the therapeutic range in the short term [17]. In contrast, a French study could show that an education program lowers the complication rate of patients without PSM [18]. An Italian study assessed the acceptance of an educational program for patients with OAT but not its effectiveness [19].

Based on these studies, our trial is the first to evaluate the long-term effects of a complex practice nurse-based patient education program in comparison to a patient brochure only.

## Hypothesis

A practice nurse-based, individual and video-assisted patient education program effectively increases long-term basic knowledge about OAT. Patients participating in this complex education program will spend more time in the therapeutic range and will have fewer complications in comparison to the control group.

## Aim

The aim of this trial is to evaluate the long-term effects of a complex patient education program about OAT in comparison to a patient information brochure only.

## Objectives

▪ To investigate long-term effectiveness (6 months) of a complex patient education program about OAT on patient knowledge

▪ To assess the effect of a complex patient education program about OAT on time spent in therapeutic range

▪ To investigate long-term effectiveness (6 months) of a complex patient education program about OAT on subjective feeling of safety

▪ To assess effect of a complex patient education program about OAT on complications related to OAT

## Method

**Figure 1:**
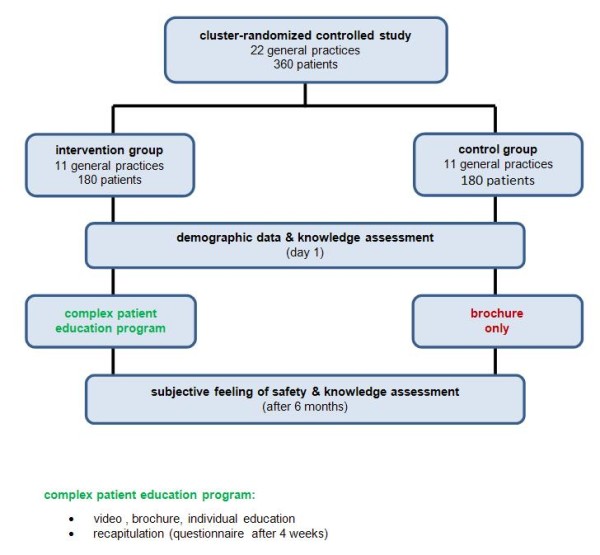
**Flow-chart**. The flow-chart gives an overview about the trial.

This trial will be a cluster-randomized controlled educational trial consisting of 22 general practices (GPs) which are expected to recruit 360 patients with OAT. Practices will be randomized into an intervention group or a control group (Figure 1).

We obtained a Universal Trial Number (UTN: U1111-1118-3464) and registered this trial in the German Clinical Trials Registry (DRKS00000586).

## Patient recruitment

### General practitioners

Monitoring and dose adjustment for OAT is managed by general practitioners in Germany. GPs in central Germany (Göttingen, Braunschweig and the surrounding areas) will be invited to participate in the trial by mail. Addresses have been obtained from the local health boards. In case of non-response to the letter, GPs will be contacted by telephone or personally. From previous experience, it seems likely that roughly 15 - 20% will agree to participate [20].

### Patients

In order to minimize sub-sampling bias, all patients with OAT will be identified at the beginning of the trial by searching the GPs' electronic medical records for the laboratory billing code "32015" for INR measurement. The inclusion criteria are age (> 18 years), OAT and willingness to participate in the trial. For dependent patients, both they and their caregivers (e.g. care giving relatives) must be willing to participate. Exclusion criteria include the inability to give informed consent and/or the lack of a caregiver willing to participate in the study. Also, nursing home residents, patients only seen in cross coverage, or patients with an insufficient command of the German language will be excluded. Lastly, patients managing anticoagulation with self-monitoring (PSM) will also be excluded.

During their next visit for INR-measurement age and gender of all eligible patients will be recorded. Patients will receive an information leaflet regarding the purpose and content of the study. Patients willing to participate will fill in a consent form. If there are further questions, patients, caregivers and/or practice nurses are encouraged to contact the study doctors.

## Randomization

The randomization of the GPs to the intervention or control group will be conducted externally by Department of Medical Statistics, University Medical Centre Göttingen using a computer routine (allocation ratio 1:1).

## Primary outcome

The primary outcome is the number of correctly answered questions from the 13-item OAT-questionnaire.

## Secondary outcome

Secondary outcomes are time spent in therapeutic range, subjective feeling of safety and complications related to OAT.

## Intervention

Practice nurses will attend a preparatory course, which will enable them to provide individual training sessions for patients based on the content of the video and brochure. The preparatory course for the practice nurses will include simulation (role play).

Patients in the intervention group will be shown an educational video and receive a patient information brochure. The video (20 minutes) provides information about OAT based on up-to-date scientific literature. It addresses common questions about OAT (Table 1). The patient information brochure was designed in accordance with the DISCERN criteria and complements the video [21].

**Table 1:** Content of the video


**1. Introduction**
- aim of this video
- scene: a patient newly on oral anticoagulant therapy consults her GP and has questions
**2. General information about oral anticoagulation**
- Why do I have to take oral anticoagulants?
- active agent: Phenprocoumon
- Explanation of stroke
- How long do I have to take oral anticoagulants?
- How do I notice that my coagulation not sufficiently inhibited?
**3. Nutrition**
- Do I have to follow a special diet?
- Which food contains a high amount of vitamin K (usual consumption quantity)?
- Alcohol, coffee
- Nutrition on holidays
**4. Drug-interactions & influences on OAT**
- Drug interactions, influence of non-prescription drugs (herbal drugs)
- Discontinuation or prescription of a new drugs
- Which non-prescription pain-killer is the safest in combination with oral anticoagulant therapy?
**5. Adherence**
- Monitoring
- Coagulation test, meaning of Quick- and INR-level, therapeutic range
- What should I do if I forgot a dosage?
- Recognition of critical situations and how to react (e.g. bleeding, black stool)
- In which situations should I mention that I receive oral anticoagulants? (e.g. pharmacy, dentist, injections)
**6. Summary of the main points**

Practice nurses will use the brochure as a guide to discuss knowledge gaps about OAT after the video in an individual training session lasting about 20-30 minutes. The intervention group has the option of another individual training if necessary. After 4 to 6 weeks when the next blood work is done, patients will be asked to fill in a short knowledge assessment questionnaire, designed to recap what was learned in the video and individual training session.

## Control

The control group will only receive the patient information brochure.

## Data collection

Demographic data and knowledge about OAT will be collected at baseline (Additional file 1). After six months, knowledge about OAT and the subjective feeling of safety in both groups will be assessed (Figure 1). The self-developed questionnaire is based on the content of the video and the information brochure. The questionnaire was piloted with 12 patients previously and optimized accordingly.

At the end of the trial after 6 months we will collect data about complications related to OAT and the INR results from patient records in both groups.

## Data safety and privacy

All data will be handled according to the Medical Confidentiality Rules and German Federal Data Security Law. Patient names and other confidential information will be coded to disable the tracking of individual patients. Informed consent and questionnaires will be mailed separately.

## Ethics

The trial is planned and will be conducted in accordance to the medical professional codex and the Helsinki Declaration of 1996 as well as the German Federal Data Security Law. The study protocol was approved by the ethics committee of the University of Göttingen (ethic committee University Medical Centre Göttingen, protocol number 2/9/10). Patients receive written and oral information about the flow of the study, its aims, potential benefits and risks prior to their consent and participation in the trial. If they agree to participate, they sign the informed consent sheet. Patients are informed that they may withdraw from the study at any time without disclosing reasons and without negative consequences for their medical care.

## Statistical analysis

We expect a total number of 22 practices which will be randomized to the two intervention arms. Assuming an intra-cluster correlation coefficient of 0.05 [22], 15 patients per practice (i.e. 22 × 15 = 330 patients in total) will give a power of at least 80% at a two-sided significance level of 5% if the standardized intervention effect is at least 0.41, which is considered a medium effect size [23]. Accounting for patient dropout, we plan to recruit a total of 360 patients, i.e. about 16 patients per practice. We do not expect any practices to drop out.

Baseline and follow-up data will be presented using standard descriptive statistics and graphs. Allowing for correlation the primary endpoint will be modelled in a mixed linear model with intervention, baseline OAT knowledge and, if imbalances between the groups occur, possibly other factors as fixed effects and a practice effect as a random cluster effect. Main interest is in testing the null hypothesis of no intervention effect. The p-value for this test will be reported alongside a 95% confidence interval for the intervention effect. Standard diagnostics will be applied to check for model fit. If model assumptions are not met, non-parametric rank-based procedures will be considered. The mixed linear model described above is to some extent robust to missing data. If the proportion of missing data is much higher than expected, alternatives will be explored [24]. Percentage time in range will be calculated according to Rosendaal's equation [25].

## Discussion

To our knowledge this will be the largest cluster-randomized controlled trial to evaluate the long-term effects of a complex patient education program about OAT in comparison to a patient information brochure only. An educational video has been used previously in a small American study (n = 22) [26]. So far, most studies only evaluated the short-term effects of education programs or assessed the acceptance of education programs for patients with OAT but not its effectiveness [17,19]. Moreover most studies were done with patients trained for self management (PSM).

Our trial will provide data about patient knowledge, the effect of education on time spent within therapeutic range and the rate of complications. Although we record complications in our study, we expect that the frequency will be too low to detect significant differences between the groups. If we can prove an increase of long-term knowledge of patients about OAT by a video-assisted education program, this program could be incorporated into routine OAT in primary care to increase patient safety.

Recruitment into this study is scheduled to the start in early 2011.

## Abbreviations

The following abbreviations have been used in the manuscript:

INR: international normalized ratio; GP: general practitioners; OAT: oral anticoagulation therapy; PSM: patient self-management.

## Competing interests

The authors declare that they have no competing interests.

## Authors' contributions

All authors contributed to the study design and the making of the educational video. Funding was obtained by JFC and SVV. TDH and JFC were the principal authors of the manuscript. All authors reviewed and approved the final manuscript.

## Pre-publication history

The pre-publication history for this paper can be accessed here:

http://www.biomedcentral.com/1471-2296/12/17/prepub

## Supplementary Material

Additional file 1 **Annex - Baseline Questionnaire**. A 13-item-questionnaire to evaluate patient knowledge about OAT at baseline (translation). Click here for file
