# Supplementary material for: Practice nursed-based, individual and video-assisted patient education in oral anticoagulation - Protocol of a cluster-randomized controlled trial
Source: BMC Fam Pract. 2011 Apr 10;12:17. doi: 10.1186/1471-2296-12-17 (PMC3089775; doi:10.1186/1471-2296-12-17)
Supplement: Additional file 1 — Annex - Baseline Questionnaire. A 13-item-questionnaire to evaluate patient knowledge about OAT at baseline (translation). [file 1471-2296-12-17-S1.DOCX]

## Annex (Baseline Questionnaire; translation)

| 1. **Why do I take oral anticoagulants?** |
| --- |
| - cardiac arrhythmia / atrial fibrillation - deep vein thrombosis - pulmonary embolism - vascular prosthesis - artificial heart valve - I do not know. |
| 1. **Which risks does the anticoagulants therapy reduce?** |
| - thrombosis/ pulmonary embolism - stroke - heart attack - I do not know |
| 1. **How long do I have to take oral anticoagulants?** |
| - several weeks - several months - several years - ever - I do not know. |
| 1. **How frequent should coagulation be controlled?** |
| - once per week - once per month - quarterly - I do not know. |
| 1. **My coagulation should be in the following range (Quick- or INR-level):** |
| Target-range: between ______ and ______.   - I do not know. |
| 1. **Which foods contain a high amount of vitamin K (usual consumption quantity)?** |
| - cabbage - potatoes - apples - green salad - tomato concentrate - spinach - onion - zucchini |
| 1. **Do I have to follow a special diet while I am taking oral anticoagulant therapy?** |
| - extra much salad and vegetables - avoid salad and vegetables - frequently salad and vegetables - I do not know. |

| 1. **Which non-prescription pain-killer is the safest while taking oral anticoagulant therapy?** |
| --- |
| - Acetylsalicylacid (z.B. Aspirin®) - Diclofenac (z.B. Voltaren®) - Ibuprofen - Paracetamol - combination-drugs (e.g. Thomapyrin®) - I do not know. |
| 1. **How long can I make up for a forgotten dosage?** |
| - up to 2 hours - up to 6 hours - up to 12 hours - up to 24 hours - never |
| 1. **How do I notice if my coagulation is not sufficiently inhibited?** |
| - joint pain - sudden headaches - diarrhea - I would not recognize it. - I do not know. |
| 1. **What could affect the effects of oral anticoagulants?** |
| - regular exercises - abdominal influenza - fever - coffee - ginkgo - fasting - moderate alcohol consumption - non-prescription medicines - I do not know. |
| 1. **When should I contact a doctor the same day?** |
| - painful swelling with or without skin discoloration - sudden speech disorder - black stool - arm weakness (also temporarily) - every incised wounds - I do not know. |
| 1. **When should I mention that I receive oral anticoagulants?** |
| - dental visits - at the drugstore or pharmacy - before injections - new prescription - before planned medical procedures (e.g. OP) - I do not know. |
